# Potentially Bioactive Fungus Mediated Silver Nanoparticles

**DOI:** 10.3390/nano11123227

**Published:** 2021-11-28

**Authors:** Abu Baker, Sana Iram, Asad Syed, Abdallah M. Elgorban, Abdullah Msaad Al-Falih, Ali H. Bahkali, Mohd Sajid Khan, Jihoe Kim

**Affiliations:** 1 Nanomedicine & Nanobiotechnology Lab, Department of Biosciences, Integral University, Lucknow 226026, India; karimabubaker@gmail.com; 2 Department of Medical Biotechnology and Research Institute of Cell Culture, Yeungnam University, Gyeongsan 38541, Korea; sanairam224@gmail.com; 3 Department of Botany and Microbiology, College of Science, King Saud University, P.O. Box 2455, Riyadh 11451, Saudi Arabia; assyed@ksu.edu.sa (A.S.); aelgorban@ksu.edu.sa (A.M.E.); aalfalih@ksu.edu.sa (A.M.A.-F.); abahakli@ksu.edu.sa (A.H.B.)

**Keywords:** AgNPS, fungus, anticancer, antibiofilm, antibacterial

## Abstract

Fungal metabolites, proteins, and enzymes have been rich sources of therapeutics so far. Therefore, in this study, the hypha extract of a newly identified noble fungus (*Alternaria* sp. with NCBI Accession number: MT982648) was used to synthesize silver nanoparticles (F-AgNPs) to utilize against bacteria, fungi, and lung cancer. F-AgNPs were characterized by using physical techniques, including UV–visible spectroscopy, zeta potential, DLS, XRD, TEM, and HR-TEM. The particles were found to be polydispersed and quasi-spherical in shape under TEM. They had an average size of ~15 nm. The well dispersed particles were found to have consistent crystallinity with cubic phase geometry under XRD and HR-TEM. The presence of different functional groups on the surfaces of biosynthesized F-AgNPs was confirmed by FTIR. The particle distribution index was found to be 0.447 with a hydrodynamic diameter of ~47 d.nm, and the high value of zeta potential (−20.3 mV) revealed the stability of the nanoemulsion. These particles were found to be active against *Staphylococcus aureus* (multidrug resistance-MDR), *Klebsiella pneumonia*, *Salmonella abony*, and *Escherichia coli* (MDR) with MIC_50_ 10.3, 12.5, 22.69, and 16.25 µg/mL, respectively. Particles also showed inhibition against fungal strains, including *A. flavus*, *A. niger*, *T. viridens*, and *F. oxysporium.* Their inhibition of biofilm formation by the same panel of bacteria was also found to be very promising and ranged from 16.66 to 64.81%. F-AgNPs also showed anticancer potential (IC_50_—21.6 µg/mL) with respect to methotrexate (IC_50_—17.7 µg/mL) against lung cancer cell line A549, and they did not result in any significant inhibition of the normal cell line BEAS-2. The particles were found to alter the mitochondrial membrane potential, thereby disturbing ATP synthesis and leading to high ROS formation, which are responsible for cell membrane damage and release of LDH, intracellular proteins, lipids, and DNA. A high level of ROS also elicits pro-inflammatory signaling cascades that lead to programmed cell death by either apoptosis or necrosis.

## 1. Introduction

In the last few decades, nanomedicines have emerged as useful tools for combatting various diseases. Due to their advantages, which include effectiveness, specificity, cost-effectiveness, safe production, and high biocompatibility, they have become a leading research topic [1]. Silver has been used worldwide for centuries as an antimicrobial agent, and nowadays silver nanoparticles (AgNPs) are used in various biomedical applications, such as targeted drug delivery systems, imaging and sensing in the field of diagnosis, and therapeutics, due to their small size and unique surface chemistry [2]. The misuse of antibiotics is leading to next-generation public health issues—such as microbes resistance to multiple drugs [3]. Due to antibiotic resistance, the treatment of bacterial infections is more complicated, as even the most advanced antibiotics can become ineffective. Therefore, AgNPs are strong candidates for bacterial treatment: they can eradicate multidrug-resistant bacteria very effectively [4]. Fungi have been used as a source of redox and stabilizing/capping agents in the green synthesis of different types of inorganic nanoparticles by virtue of generation of their varieties of molecules of medicinal importance, easy handling, and the low toxicity of their residues. Alternaria sp., Fungi Imperfecti, has been reported to produce at least 268 pharmacologically active metabolites, including nitrogen-containing compounds, steroids, terpenoids, pyranones, quinines, and phenolics, etc., that could be used in different specific medicinal or agricultural applications [5]. It also secretes phytotoxins and mycotoxins of medicinal importance, such as porritoxin [6], a cancer chemopreventive agent, and an inhibitor of histone deacetylase (HDAC), respectively. Furthermore, green synthesis enables particles to immobilize biomolecules of a fungus on their surfaces, which provide enhanced stability and may bestow biological activities [7]. AgNPs raise few concerns regarding toxicity towards humans [8]; however, exposure can cause a disturbance in the cell membrane, which eventually leads to leakage of cellular content or impaired mitochondrial activity [9]. However, low doses of AgNPs are known to be non-toxic and are a highly effective antibacterial agent. Therefore, they are being used in surgical prosthesis, water purifier filters, and nano-formulations [10]. Physical and chemical syntheses of metallic nanoparticles involve high temperatures, high pressures, various toxic chemicals, and complex chemical reactions, making these processes dangerous and causative of various side effects [11]. Biological or green synthesis of nanoparticles is a preferrable solution which could not only decrease or remove the use of toxic and costly chemicals, but also allow highly cost-effective and large scale production with biocompatible machines [12]. The intrinsic properties of capping phytochemicals, proteins, and enzymes make AgNPs biogenic and highly biocompatible [13]. Green synthesis of nanoparticles follows the bottom-up approach, where the oxidation and reduction reactions take place first [14]. Phytochemicals and enzymes (microbial, fungal, algal, plant, animal, human) are accountable for the exchanging of electrons between metal ions and different molecules during the production of biogenic metal nanoparticles [15]. Moreover, different types of bacteria, fungi, algae, and plants, and their extracts are also well known for their toles in the biosynthesis of AgNPs [16,17]. Biosynthesized AgNPs have been demonstrated to be antimicrobial, antifungal, antialgal, antipathogenic, and anticancer agents [18]. The surface functionalization of these particles with antibiotics and antifungal agents for various applications is very effectively compared to using pure antibiotics and antifungals [9]. The different types of mechanisms behind the action of AgNPs against bacteria are ATP production hindrance, protein synthesis inhibition, DNA replication disruption, reactive oxygen species (ROS) generation, reduction of glutathione level, and the most common, cell membrane disruption [19].

In this study, an aqueous hypha extract of the newly identified noble *Alternaria* Sp. (isolated from the medicinal plant garden, department of pharmacy, Integral university, Lucknow) was used to synthesize biogenic F-AgNPs. Eventually, these particles were characterized by different physical techniques. The extract facilitated a reduction (from Ag^+^ to Ag^0^) by generating the required redox potential via the synergistic effect of different metabolites present in the extract, and capping of the particles, which not only provided the stability but also made them biocompatible in nature. The biogenic NPs imitated the properties of the metabolites and proteins/enzymes of the extract. Further, these F-AgNPs showed antibiofilm and antimicrobial properties against MDR *Staphylococcus aureus* (NCIM 2079), MDR *Escherichia coli* (NCIM 2571), *Klebsiella pneumoniae* (ATCC 13883), and *Salmonella abony* (ATCC 6017). They also showed antifungal activity against *Aspergillus flavus* (MTCC 1475), *Aspergillus niger* (ITCC 1617), *Trichoderma viridens* (MTCC167), and *Fusarium oxyporium* (NCIM No. 1008). F-AgNPs were also found to have anticancer properties against the lung cancer cell line A549, yet they did not have any significant toxic effects against a normal human lung cell line (BEAS-2).

## 2. Material and Methods

### Material

All chemicals were of analytical grade and procured from Sigma and Hi-Media Mumbai, India. For buffer and media preparation Ultrapure Milli-Q water was used.

## 3. Methods

### 3.1. Aqueous Extract of Fungal Hyphae

First, 10 g of isolated fungus (*Alternaria* sp. with NCBI Accession number: MT982648) biomass was washed with 100 mM of Tris-buffer and crushed in liquid nitrogen repeatedly, unless fine powder was formed, and then suspended in 5 mL of Tris-buffer for 30 min. Eventually, the freeze and thaw process was repeated twice with 2 min of incubation at 4 °C on ice. Finally, the suspension was sonicated for 5 min and the supernatant which contained aqueous extract was concentrated using a rotavapour. The protein estimation of the final concentrate was estimated by the Bradford method.

### 3.2. Qualitative Analysis of Phytochemicals

The qualitative analyses of phytochemicals of aqueous extract of fungal hyphae were performed as per standard procedures [20].

### 3.3. In Vitro Biological Synthesis of Aqueous Fungal Hypha Extract-Mediated F-AgNPs

The synthesis of F-AgNPs was performed by incubating the isolated hypha extract (containing 1 mg protein) with the suspension of 1 mM of AgNO_3_ in 3 mL of double-distilled water and incubated for 7 days at 40 °C. The incubation period played a decisive role in the synthesis of nanoparticles. The synthesis of F-AgNPs was monitored as per the change in the color of the solution, and it was further confirmed by UV–visible spectroscopy with λ_Max_ at 422 nm. The unbound salt, impurities, metabolites, and proteins/enzymes were isolated with 50% ethanol treatment [21]. 

### 3.4. Characterization of Biosynthesized F-AgNPs

The progress of the synthesis of F-AgNPs was monitored with a UV–vis spectrophotometer (Shimadzu dual-beam spectrophotometer, model UV-1601 PC Tokyo, Japan). This was operated at a resolution of 1 nm in a quartz cuvette. The absorption spectra of F-AgNPs in colloidal suspension were gathered in the range of 200 to 800 nm. Milli-Q water was taken as a blank. The size of the inorganic core, the 2D internal structure, and images from high-resolution transmission electron microscopy were analyzed by transmission electron microscopy (JEOL, JEM-2010 GatanDigital, Hillsboro, OR, USA) operated at an acceleration voltage of 200 kV. The grid was prepared by loading a drop of synthesized F-AgNPs on a carbon-coated copper grid and incubating it in infra light for 25 min to evaporate the solvent. The mean particle size (hydrodynamic diameter) was determined by dynamic light scattering (DLS) using a zeta sizer (Malvern Instrument Ltd, Malvern UK). Zeta potential estimation was done by considering the Smoluchowski approximation with the help of the electrophoretic mobility values in an aqueous solution. Powder XRD diffraction patterns were recorded using a PAN Analytical instrument equipped X’celerator (Malvern Panalytical Ltd Malvern WR14 1XZ United Kingdom), a fast solid-state detector on the drop-coated sample on the glass substrate. The sample was scanned using X’celerator with a total number of active channels of 121. Iron-filtered CuKα radiation (λ = 1.5406 Å) was used. XRD patterns were recorded in the 2θ range of 20°- 70° with a step size of 0.02° for 5 s per step. FTIR of F-AgNPs was performed by preparing a film by placing a drop of the F-AgNPs solutions on a Si (111) substrate, and evaporating of water was carried out by delicate warming. A Perkin-Elmer Spectrum FTIR system (PerkinElmer Inc., Waltham, MA, USA) apparatus worked in the disperse reflectance style at a declaration of 4 cm^−1^. To get great signal-to-noise proportions, 256 outputs of F-AgNPs film were taken in the range 400–4000 cm^−1^.

### 3.5. Antibacterial and Antifungal Activities of F-AgNPs

The antibacterial and antifungal potential of biosynthesized F-AgNPsand fungus isolated protein mixture (P-mixture) were evaluated against a panel of bacteria having *Staphylococcus aureus* (MDR), *Klebsiella pneumonia*, *Salmonella abony*, and *Escherichia coli* (MDR) and an another panel of fungi including *Aspergillus flavus*, *Aspergillus niger*, *Trichoderma viridens*, and *Fusarium oxyporium* by using agar well diffusion method. In the given procedure,25 mL of nutrient agar (for bacteria) and potato dextrose agar (for fungi) was poured into the sterilized petri dishes and eventually 120 μL of each freshly prepared bacterial and fungal broth culture (1 × 10^4^ CFU/mL) was used for bacterial and fungal lawn preparation, respectively. The 10 mm diameters of agar wells were cut with the help of a stainless steel cork borer, and 75 μL of F-AgNPs extract and 75 μL of isolated aqueous fungal hypha extract (protein concentration/P-mixture—333 µg/mL) and a milli-Q as a control were loaded in the prepared wells. The final prepared plates were incubated for 24 h at 37 °C (for bacteria) and for 48 h at 27 °C (for fungi) subsequently, the zones of inhibition were measured in mm.

### 3.6. Determination of Minimum Inhibitory Concentration (MIC)

The antibacterial potential of F-AgNPs was calculated by their MICs against *Staphylococcus aureus* (MDR), *Klebsiella pneumonia*, *Salmonella abony,* and *Escherichia coli* (MDR). The bacterial strain was collected in the mid-logarithm phase and diluted to 2 × 10^5^ CFU/mL in 0.03% of Luria-Bertani (LB) broth in PBS. The 100 μL of LB medium consist of F-AgNPs were serially diluted in 96 well plates [22]. The lowest concentration of F-AgNPs at which 25, 50, 75% growth of microbes was inhibited is defined as MIC_25_, MIC_50,_ and MIC_75_, respectively. For negative control, Milli-Q water was used for each experiment.

### 3.7. Anti-Biofilm Potential of F-AgNPs

The extent of antibiofilm potential of F-AgNPs was determined by double dilution method [23]. In this experiment, each well of the microtiter plate was supplied with 190 μL of nutrient broth media and 10 μL of freshly prepared bacterial culture of *Staphylococcus aureus* (MDR), *Klebsiella pneumonia*, *Salmonella abony* and *Escherichia coli* (MDR). Eventually, the MIC_25_, MIC_50,_ and MIC_75_ concentration of F-AgNPs were added to each well of plate and incubated at 37 °C for 24 h. Further, the content of each well is discarded and washed twice with PBS (pH 7.2) to evacuate planktonic bacteria. The biofilms produced by adherent sessile bacteria at the walls of corresponding wells were fixed by sodium acetate (2.5%, *w/v*) and crystal violet dye (0.25%, *w/v*) was used to stain the biofilm. The wells were rinsed with Milli-Q water to remove the excess stain and incubated at room temperature for drying. Subsequently, 100 μL of DMSO was added to each dried well, and we measured the absorbance at 620 nm on ELISA plate reader (Multiskan^®^ EX, Thermo Scientific, Vantaa, Finland). The absorbance (O.D.) was considered directly proportional to the amount of biofilms produced by the corresponding adhering bacteria around the walls of the well. The percent biofilm inhibition was calculated by following the given Equation (1). Each experiment was performed in triplicate and the F-AgNPs free cultures of each bacterium served as a positive control.

(1) $$\mathrm{Percent}\;\mathrm{biofilm}\;\mathrm{inhibition}=1-\left(\frac{OD_{620}ofF-AgNPstreatedcells}{OD_{620}ofnontreatedcontrolcells}\right)\times 100$$

### 3.8. Cell Culture and Maintenance

The human non-small cell lung cancer (NSCLC) cells and BEAS-2 cells were procured from National Centre for Cell Science (NCCS), Pune, India. The A549 cells were grown in DMEM-F12 whereas BEAS-2B cell line was seeded in α-MEM medium and both were supplemented with 1.5% antibiotic-antimycotic solution that consists of penicillin, streptomycin, and amphotericin B (Himedia, India, Ltd., Mumbai, India) and 13% FBS (fetal bovine serum) solutions in humified incubator at 37 °C temperature and 5% CO_2_ environment.

### 3.9. Determination of Cytotoxicity by MTT Assay

The cytotoxic efficacy of biologically synthesized F-AgNPs and pure drug methotrexate (MTX) against A549and BEAS-2B cells were evaluated by seeding a 1 × 10^5^ cells per well in a 96 well plate and incubated in 5% CO_2_ humidified incubator at 37 °C for 24 h. After confluency, the cells were treated with F-AgNPs and MTX at concentration of 2.5, 5, 7.5, 10, 12.5, 15, 17.5, 20, 22.5, and 25 μg/mL and again incubated for 24 h. Subsequently, 20 μL (5 mg/mL in PBS) of MTT [3-(4,5-dimethylthiazol-2-yl)-2,5- diphenyl-tetrazolium bromide] was added in each well and incubated for 2 h at 37 °C in 5% CO_2_ incubator. After the incubation, the media of wells were discarded and the resulting formazan crystals were dissolved in 80 μL of DMSO. By using a microplate reader (BIORAD-680 California, USA) at 570 nm with a reference filter of 655 nm the reduced MTT was quantified and by using Equation (2) the inhibition percent of the cells were calculated [24].

(2) $$Percent\;inhibition=100-\left(\frac{Atest-Ablank}{Acontrol-Ablank}\right)\times 100$$

where *Atest*—sample treated absorbance, *Ablank*—blank absorbance, and *Acontrol*—control sample absorbance. The results are demonstrated as percentages of cells inhibited as compared to the control.

### 3.10. Determination of Reactive Oxygen Species (ROS) Generation

Generation of ROS in F-AgNPs-treated A549 viable cells was identified by staining with 5- and 6-carboxy-2′,7′-dichlorodihydrofluorescein diacetate (DCFDA) dye. The non-specific intracellular esterase produced by generated ROS during oxidative stress in the cells deacetylates non-fluorescent DCFDA dye and emits bright green fluorescence due to oxidation of reduced fluorescein compound [25]. The A549 cells of density 3 × 10^5^ cells/well were seeded in the 24 well plates and incubated for 24 h at 37 °C in a 5% CO_2_ incubator and then treated with an IC_50_ concentration of F-AgNPs, and again incubated further for 24 h. Eventually, 10 µM of DCFDA dye was added in each well and they were incubated for 30 min at 37 °C. After incubation, the reaction solutions were discarded and 200 µl of PBS was added in each well. The intracellular fluorescence of cells was analyzed by using an inverted fluorescence microscope (Nikon ECLIPSE Ti-S, Tokyo, Japan).

### 3.11. Detection of Changes in Nuclear Morphology by DAPI Staining

The nuclear fluorescent dye DAPI was used to investigate the apoptotic effect of biologically synthesized F-AgNPs on A549 cells. The A549 cells at a density of 3 × 10^5^ cells/well were seeded in the 24 well plates and incubated for 24 h at 37 °C in 5% CO_2_ incubator and then treated with an IC_50_ concentration of F-AgNPs and again incubated further for 24 h. After incubation 4% paraformaldehyde was added for 10 min to fix the cells, followed by permeabilizing buffer (3% paraformaldehyde and 0.5% Triton X-100) for permeabilization. After permeabilization, cells were stained with 300 nm DAPI dye for 15 min and then images were obtained under the fluorescence microscope (Nikon ECLIPSE Ti-S, Tokyo, Japan).

### 3.12. Determination of Mitochondrial Membrane Potential (ΔΨm)

Mito Tracker Red CMX Ros staining was used to estimate the mitochondrial membrane potential in F-AgNPs-treated A549 cells. Cells at a density of 3 × 10^5^ A549 cells/well were grown in 24 well plates and allowed to adhere properly and treated withIC_50_ concentration of F-AgNPs. Finally, the treated cells after 24-h of incubation were stained with Mito Tracker Red dye (300 nM) and further incubated for 30 min in dark. The images of stained cells were captured under an inverted fluorescence microscope (Nikon ECLIPSE Ti-S, Tokyo, Japan).

### 3.13. Statistical Analysis

All statistical analyses were performed using the Origin 6.0 software (USA), as described previously [26].

## 4. Results and Discussion

### 4.1. Biological Synthesis of F-AgNPs and Characterizations

**Figure 1:**
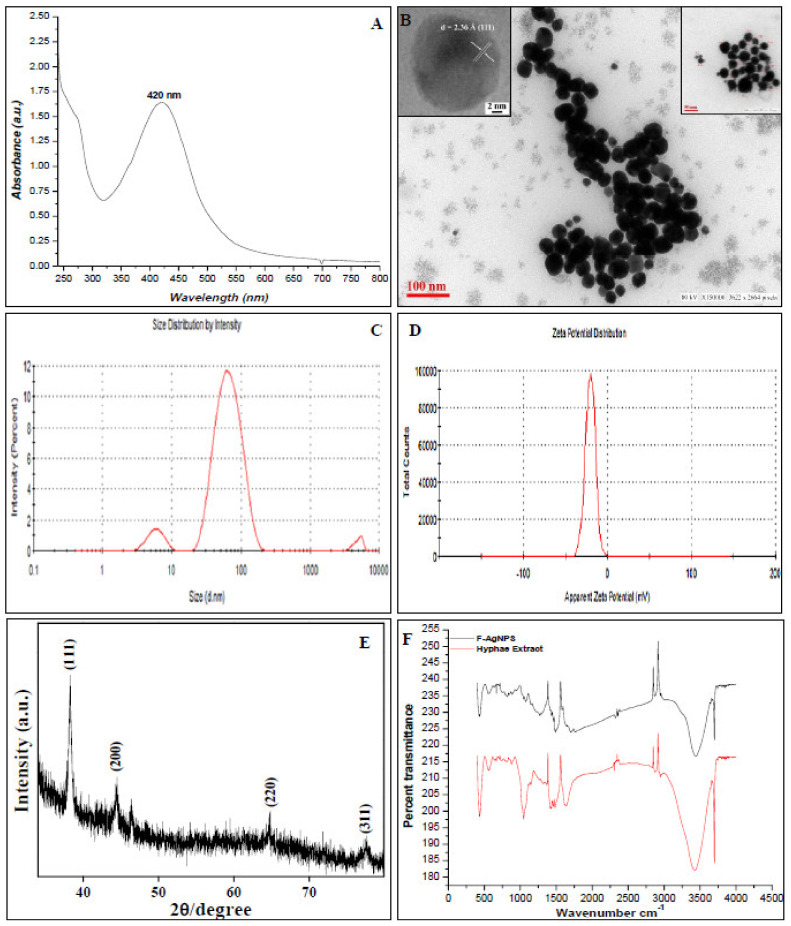
The physical characterization of F-AgNPs by (**A**) UV–visible spectroscopy (λ_Max_ 420 nm); (**B**) transmission electron microscopy (average size ~15 nm) HR-TEM and higher magnification in the insets; (**C**) dynamic light scattering (average size 47 d.nm); (**D**) zeta potential (−20.3 mV); (**E**) XRD and (**F**) FTIR analyses.

Aqueous hypha extract of noble *Alternaria* Sp. was used to synthesize biogenic F-AgNPs. The peak (λ_Max_ at 422 nm) consequent to surface plasmon resonance (SPR) of silver nanoparticles verified the synthesis of the given nanoparticles (Figure 1A) [27]. The polydispersity without agglomeration and spherical shape of F-AgNPs were revealed by the TEM micrograph. The average size of F-AgNPs was found to be 15 ± 1 nm after TEM analysis (Figure 1B). The extent of single-crystallinity under HR-TEM analysis (Figure 1B, Left Inset) was found to be quite remarkable in the as-synthesized particles in ambient conditions. The lattice planes exhibited a spacing of ~2.36 Å for the given Ag nanoparticles for the lattice planes {111} with cubic phase geometry. The hydrodynamic diameter of F-AgNPs was 47 nm (Figure 1C) with a particle distribution index of 0.447, and they were found to be highly stable with a zeta potential of −20.03 mV (Figure 1D). To further verify the phase purity and crystallinity of Ag nanoparticles using X-ray diffraction analysis, the X-ray diffraction patterns were recorded from drop cast films of F-AgNPs on a glass substrate (Figure 1E), showing intense peaks corresponding to planes {111}, {200}, {220}, and {311}. The peak position and 2θ values concur with those reported for Ag nanoparticles. Almost all peaks in the pattern could be indexed to cubic Ag nanoparticles with cell parameters of a = b = c = 4.086 and α = β = γ = 90°, which are close to those reported in the literature. The XRD patterns showing peak broadening in XRD spectra suggest that particles were formed in the nanosized regime. To calculate the average particle size of Ag nanoparticles, the Scherrer equation was used.

B = K λ/bcos θ

where λ is the X-ray wavelength, B is the average size, θ is the diffraction angle, b is the full width half maximum intensity (FWHM), and K∝, a constant, is usually equal to 1. The average particle size of F-AgNPs under XRD analysis was found to be ~11 nm.

FTIR measurements were accomplished to identify the presence of various functional groups in the biomolecules responsible for the bio-reduction of Ag^+^ and capping/stabilization of silver nanoparticles (Figure 1F). The band at 3472 cm^−1^ in the spectrum corresponds to O-H stretching vibration indicating the presence of alcohol and phenol. The band at 2912 cm^−1^ and the 2867 cm^−1^ region arising from C-H stretching of the aromatic compound were observed. The band at 1768 cm^−1^ was assigned for (C-C) stretching (non-conjugated). The band at 1642 cm^−1^ in the spectrum corresponds to C-N and C-C stretching, indicating the presence of protein. The band at 1450 cm^−1^ was assigned to N-H stretching vibrations present in the amide linkages of proteins. These functional groups have a role in the stability/capping of Ag NPs. The bands at 1450 and 1062 cm^−1^ were assigned to N-H and C-N (amines) stretching vibrations of proteins, respectively.

FTIR spectrum of plant aqueous extract: the bands at 3429, 2931, 2861, 1631, 1436, 1342, and 1124–911 cm^−1^ correspond to polyphenols, carboxylic acids and their derivative (C=O), N-H stretching, and C=N stretching of aliphatic amines.

### 4.2. Antibacterial and Antifungal Potential of F-AgNPs

**Figure 2:**
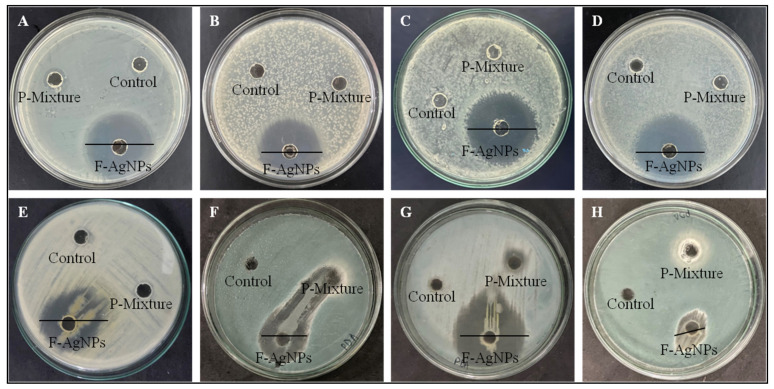
Images showing the antibacterial zones of inhibition of F-AgNPs and the fungus isolated protein mixture (P-mixture) against (**A**) MDR *Staphylococcus aureus*, (**B**) *Klebsiella pneumonia*, (**C**) *Salmonella abony,* (**D**) MDR *Escherichia coli* bacteria and antifungal zone of inhibition against (**E**) *Aspergillus flavus*, (**F**) *Aspergillus niger*, (**G**) *Trichoderma viridens*, and (**H**) *Fusoriumoxyporium* fungus. Milli-Q water was used as a control.

**Figure 3:**
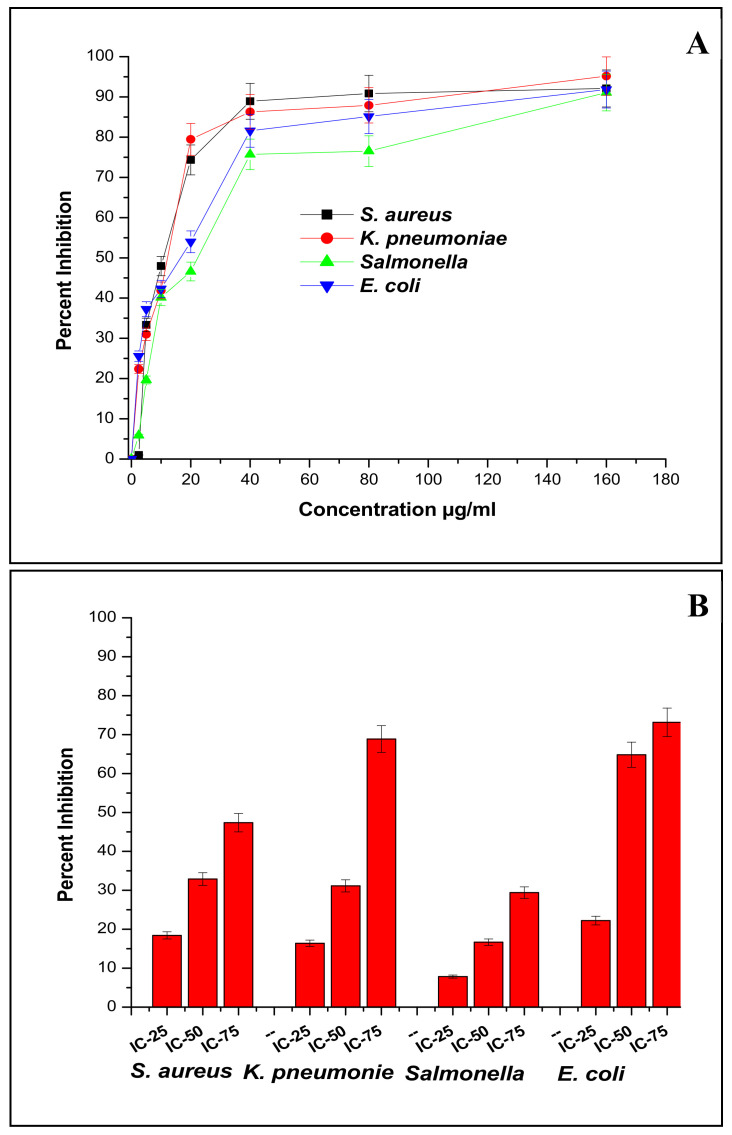
Graph showing (**A**) antibacterial potential by minimum inhibitory concentration (MIC) of F-AgNPs. Aliquots of F-AgNPs were serially diluted in 96-well microtiter plates in tryptic soy broth (TSB) medium. (**B**) Percentages of antibiofilm inhibition of F-AgNPs against MDR *Staphylococcus aureus*, *Klebsiella pneumonia*, *Salmonella abony,* and MDR *Escherichia coli*. Every datum shown is the mean ± SD of three experiments.

The intrinsic antibacterial potential of silver nanoparticles is well known, and their mode of action has been explained broadly through direct and ion-mediated destruction mechanisms [28] Generally, AgNPs are found to be more active against Gram-negative bacteria than Gram-positive due to differences in the cell wall structures of these microbes [29]. However, some biogenic AgNPs contradict this finding or show variable effectiveness among bacterial groups [30]. The antibacterial action of F-AgNPs against various bacteria was found to be satisfactory. They produced zones of inhibition against *Staphylococcus aureus* (MDR), *Klebsiella pneumonia*, *Salmonella abony*, and *Escherichia coli* (MDR) of 3, 2.9, 3.1, and 3.2 mm, respectively (Figure 2A–D). Their antifungal zones of inhibition against *Aspergillus flavus*, *Aspergillus niger*, *Trichoderma viridens*, and *Fusorium oxyporium* were 2.3, 2.1, 2.5, and 1.1 mm, respectively (Figure 2E–H). Further, their MIC_50_ values against *Staphylococcus aureus* (MDR), *Klebsiella pneumonia*, *Salmonella abony*, and *Escherichia coli* (MDR) were 10.3, 12.5, 22.69, and 16.25 µg/mL, respectively (Figure 3A), suggesting a broad-spectrum effect. The bactericidal effect of F-AgNPs is due to their adherence to the surfaces of bacterial cell walls and interactions with sulfur-containing proteins. They cause irreversible changes in sulfur-containing proteins’ structures [31]; reduce the compactness of lipid bilayer; and alter the permeability of the cell membrane, which leads to leakage of cellular contents, including ions, proteins, sugars, and the cellular energy reservoir [32]. F-AgNPs were found active against a panel of G^+^ and G^−^ bacteria due to the presence of antibacterial secondary metabolites of *Alternaria* sp., including dibenzopyranones and its derivatives, tenuazonic acid, altechromone A, altenusin, alternariol, alternariol monomethylether, altertoxin I, altertoxin II, and alterperylenol [33]. Furthermore, F-AgNPs act against bacteria through excessive generation of reactive oxygen species—H_2_O_2_, O_2_^−^, OH^.^, etc.—which interfere with respiration and membrane transport, release potassium ions from these cells, and interfere with cellular growth [34]. They also cause apoptosis-like responses; lipid peroxidation; depletion of antioxidant enzymes, such as GSH; and DNA damage [35]. These particles and silver ions can damage cellular structures (e.g., ribosomes), and biomolecules such as proteins, lipids, and DNA, by interacting with them, and hence, disturb their maintenance and activities. Inhibition of ATP synthesis is one of the strategies of AgNPs [36] against *S. aureus* and *P. aerogenosa*. The antifungal potential of F-AgNPs is due to their smaller size which facilitates their penetration easy into fungal cell walls. They then interact with the cell membrane, which facilitates their internalization into the cells. The unique intrinsic surface properties of F-AgNPs lead them to them reacting with cellular components. Generally, silver ions (released from F-AgNPs) and F-AgNPs can quickly bind to thio-enzymes/proteins, deactivate some of the house keeping enzymes, disturb the proton pumps, and make transport nonspecific across the cell membrane through interfering with membrane proteins or the phospholipid bilayer. The leakage of different ions, including H^+^, causes fungal cell disruption. Similarly, the particles can also disrupt the natural architecture of DNA by cross-linking with DNA bases, combining with DNA bases to form cross-links, and substituting the hydrogen bonds adjoined to nitrogen in purines and pyrimidines. The altered fungal DNA would be unable to replicate and become the cause of fungal death [37].

### 4.3. Antibiofilm Potential of F-AgNPs

Biofilms mainly consist of exopolysaccharides (an essential component of biofilms), nucleic acids, and proteins. They can be made by single or multiple bacterial species [38]. The formation of a bacterial biofilm is initiated by an environment where the feasibility of synthesis of exopolysaccharides (EPS) is greatly favored. Therefore, inhibition of biofilm formation could be achieved by restricting the synthesis of EPS by F-AgNPs. Most microbial infections (65–80%) are caused by biofilm forming bacteria. The immense resistance in biofilm-forming bacteria is due to the alterations in the antimicrobial enzymes, development of drug resistance properties, and non-permeability towards antimicrobial drugs [39]. *K. pneumoniae,* one of the prominent biofilm forming bacteria, uses *treC*, *sugE*, and AcrAB pump genes [40], along with type 1/3 fimbriae, lipopolysaccharides, and outer membrane proteins to evade the immune system during infection and to establish a biofilm [41]. Hence, F-AgNPs might inhibit efflux pumps to disrupt biofilm formation [42]. *S. aureus* produces multilayered biofilms in which it becomes entrenched within a glycocalyx or slime layer with clusters of heterogeneous proteins. It produces a polysaccharide intercellular antigen through SrrAB (respiratory response regulator) in anaerobic conditions via binding of a 100 bp DNA sequence upstream of the *icaADBC* operon [43]. Such types of biofilms would be inhibited by F-AgNPs disrupting the glycocalyx. The formation of a *Salmonella* biofilm is the outcome of *AgfD*, *AgfB* (encoding Tafi or curli thin aggregative fimbriae), and *AdrA* (produces cellulose). The thin aggregative fimbriae facilitate the bacterium adhering and colonizing surfaces [44]. Similarly, fim (type 1 fimbriae or pili) of *E. coli* provides attachments to abiotic surfaces. The expression of *fim* is elicited by adhesion and initial development of a biofilm. *csg* produces curli fimbriae, which maintain strong attachments with abiotic surfaces through extracellular matrix proteins. This enhances cell-to-cell communication [45]. Other essential contributing genes for biofilm formation are csgD, hha, the bcsA operon, pgaC, fimB, AcrB, and AcrEF [46].

F-AgNPs had some properties from the hypha extract due to tenuazonic acid and altechromone A which are excellent inhibitors of biofilm formation by Gram-positive strains (70–80% inhibition) and moderate inhibitors against biofilm formation by Gram-negative strains (40–60%). Tenuazonic acid, altenusin, and cyclic dipeptides also exhibited biofilm inhibition properties through quorum sensing against both G^+^ and G^−^ bacteria [33]. Biosynthesized F-AgNPs showed their potential against the biofilm formation of *Staphylococcus aureus* (MDR), *Klebsiella pneumonia*, *Salmonella abony*, and *Escherichia coli* (MDR). The biofilm inhibition was found to be dose dependent, and with an increase in the concentration of F-AgNPs, the formation of biofilms decreased [47]. The F-AgNPs used at MIC_25_, MIC_50,_ and MIC_75_ against *Staphylococcus aureus* (MDR) biofilm formation reduced it by 18.42%, 32.89%, and 47.36%; reduced *Klebsiella pneumonia* biofilm formation by 16.39%, 31.14%, and 68.85%; reduced *Salmonella abony* biofilm formation by 7.8%, 16.66%, and 29.41%; and reduced *Escherichia coli* (MDR) biofilm formation by 22.22%, 64.81%, and 73.14% (Figure 3B). In general, silver nanoparticles do have antibiofilm activity; and F-AgNPs along with bioactive capping agents, acted synergistically against biofilm formation. The antibiofilm potential of F-AgNPs depends on various factors, such as the size and shape of nanoparticles, which affect the penetration limit, and also depends on the affinity between the F-AgNPs and biofilm [47]. The above result significantly validated that F-AgNPs have antibiofilm potential against biofilms produced by MDR *Staphylococcus aureus*, *Klebsiella pneumonia*, *Salmonella abony*, and MDR *Escherichia coli*. This means that AgNPs are biofilm distracting agents.

### 4.4. In Vitro Anticancer Analysis of Biologically Synthesized F-AgNPs

#### Cytotoxicity Analysis of F-AgNPs and Its Morphological Changes in A549 Cells

**Figure 4:**
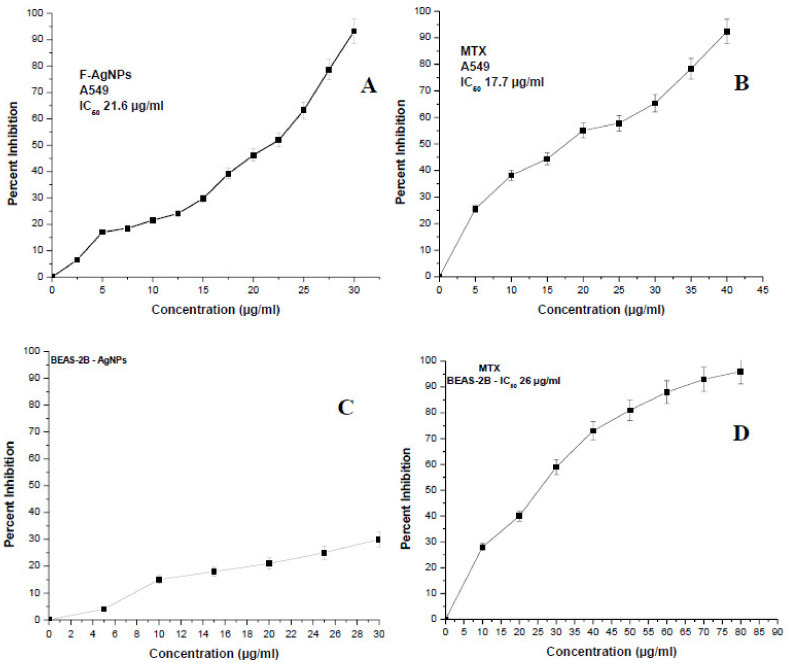
The dose-dependent cytotoxicities of (**A**) F-AgNPs treated A549 cells, (**B**) methotrexate (MTX) treated A549 cells, (**C**) F-AgNPs treated BEAS-2B cells, and (**D**) methotrexate (MTX) treated BEAS-2B cells, were evaluated after 24 h of incubation at 37 °C via MTT assay. Every datum shown is the mean ± SD of three experiments.

Biogenic F-AgNPs and MTX were analyzed at various concentrations against A549 and BEAS-2 cells. The inhibitory activities of F-AgNPs and MTX against A549 cells were found to be dose-dependent. The inhibition of A549 cells increased with an increase in the concentration of F-AgNPs or MTX, and the IC_50_s were determined to be 21.6 and 17.7 µg/mL, respectively (Figure 4A,B). F-AgNPs did not have any significant toxic effects against BEAS-2 (Figure 4C), whereas the pure drug MTX had a significant anti proliferation effect on BEAS-2 cells and was found to have an IC_50_ equal to 26 µg/mL (Figure 4D).

**Figure 5:**
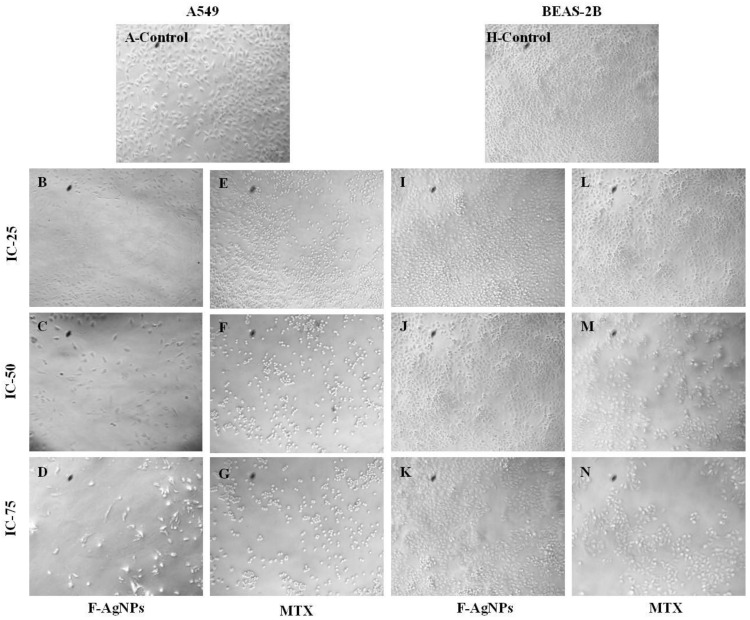
Cytomorphological images of A549 and BEAS-2 cells after 24 h in MTT assays: (**A**) untreated A549 cells; (**B**–**D**) F-AgNPs-treated A549 cells at IC_25_, IC_50_ and IC_75_, concentrations, respectively; (**E**–**G**) MTX-treated A549 cells at IC-25, IC-50, and IC-75 concentrations, respectively; (**H**) untreated BEAS-2 cells; (**I**–**K**) F-AgNPs-treated BEAS-2 cells at IC-25, IC-50, and IC-75 concentrations, respectively; (**L**–**N**) MTX-treated BEAS-2 cells at IC-25, IC-50, and IC-75 concentrations, respectively.

The anticancer potential of F-AgNPs is due to several secondary metabolites of *Alternaria* sp.: alternariol and sulfated derivatives of alternariol and its monomethyl ethers. Alternariol 5-O-methyl ether, altenusin, desmethylaltenusindesmethylaltenusin, alterlactone, and altertoxin I are prominent cytotoxic compounds. They prevent cell proliferation through inhibiting different protein kinases [48]. The internalization of the particles is largely a charge and size dependent phenomenon. Generally, most cellular internalization of particles take place through receptor (clathrin or caveolae)-mediated endocytosis, whereas a small amount is internalized through diffusion (translocation) [49]. These particles can reduce the mitochondrial membrane potential after accumulating in them, which disrupts ATP synthesis and leads to ROS formation [50]. F-AgNPs, along with their released ions, generate large amounts of ROS, which leads to oxidative stress, which is responsible for damage to the cell membrane and the release of LDH, intracellular proteins, lipids, and DNA [51]. The high level of ROS also elicits pro-inflammatory signaling cascades that lead to programmed cell death by either apoptosis or necrosis [52]. Moreover, silver ions released from F-AgNPs generate a lysosome-enhanced Trojan horse effect and inhibite cell proliferation through interacting with the membrane proteins and activating signaling pathways [53]. Moreover, phagocytosis was elicited in one study by F-AgNPs generating cytotoxicity in macrophages, which caused ROS generation and stimulated the production of tumor necrosis factor-*α*. Eventually, the cytokine created havoc among cells, including macrophages, through cell membrane damage and cellular apoptosis [54]. Significant changes were observed via morphology in A549 cells treated with F-AgNPs and MTX at IC25, IC50, and IC75 concentrations, such as loss in membrane potential, necrosis, cell proliferation inhibition, cell aggregation, and cytoplasmic condensation (Figure 5B–G) [55]. Untreated A549 cells did not show any remarkable changes in the morphology (Figure 5A). F-AgNPs-treated BEAS-2 cells did not show any substantial changes in the morphology when treated with IC_25_, IC_50_, or IC_75_ (Figure 5I–K) as compared to the control (Figure 5H). BEAS-2 cells treated with MTX at IC_25_, IC_50_, and IC_75_ experienced loss in membrane potential, necrosis, cell proliferation inhibition, cell aggregation, and cytoplasmic condensation (Figure 5L–N).

### 4.5. Detection of Intracellular ROS Generation in F-AgNPs Treated A549 Cells

**Figure 6:**
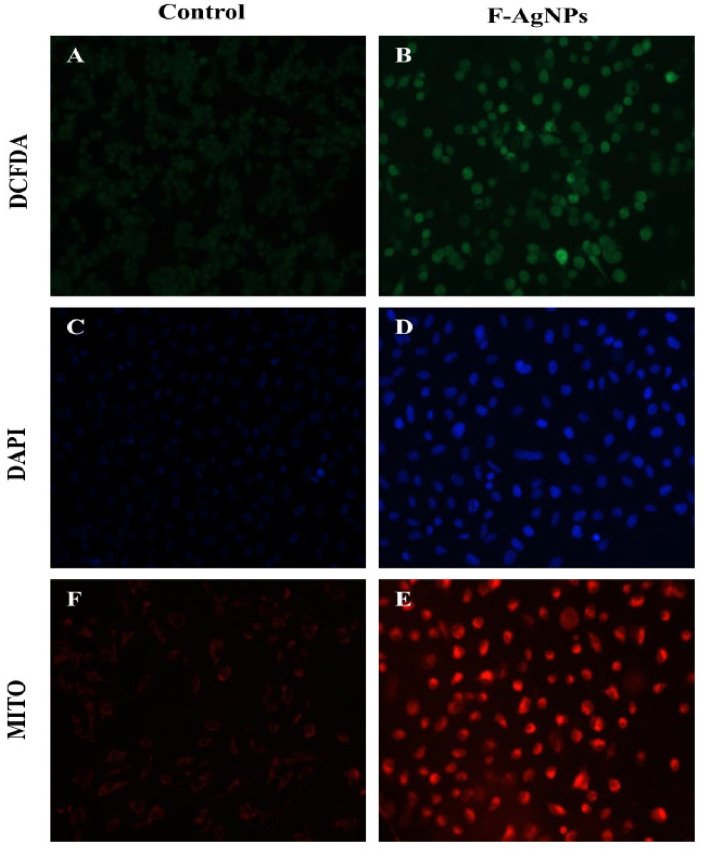
Image showing the effects of F-AgNP treatement on A549 cells at 20× magnification with ROS staining: (**A**) control and (**B**) IC-50; DAPI staining: (**C**) control and (**D**) IC-50; and Mito Tracker Red: (**E**) control and (**F**) IC-50.

**Figure 7:**
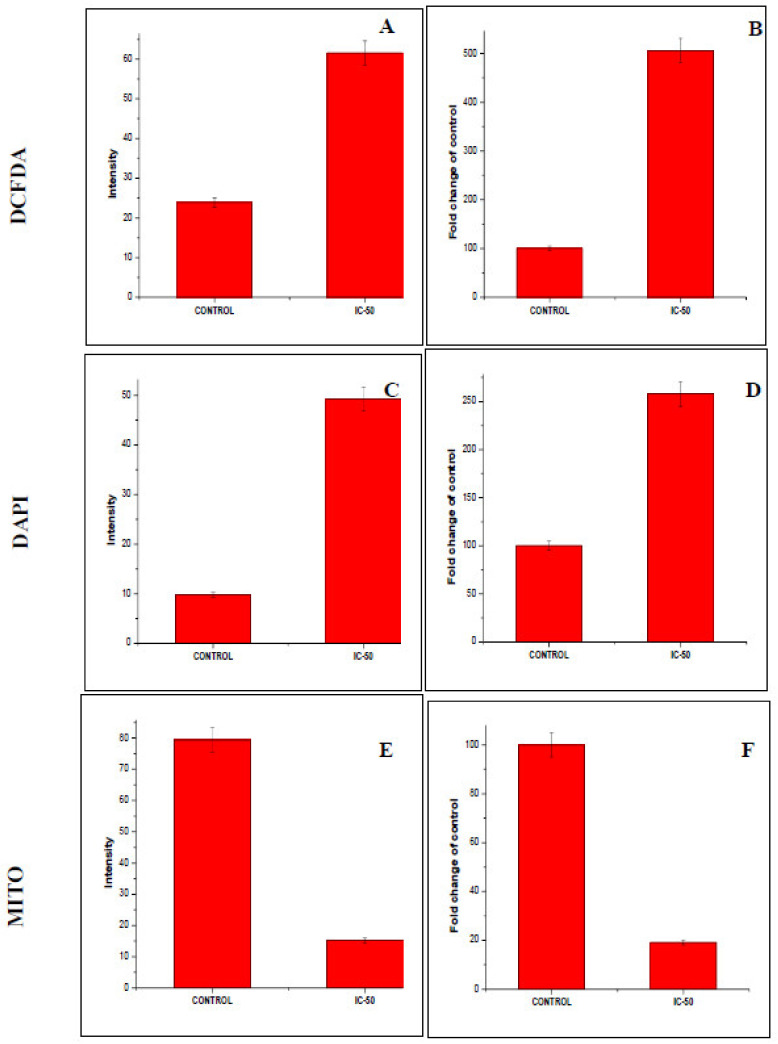
Quantification of intensity and fold changes in fluorescence—via ROS, DAPI, and MITO staining—of F-AgNPs-treated A549-treated cells at IC-50 concentration, in comparison to untreated control cells. Image J software and a fluorescent microscope were used to quantify the cellular images. (**A**) Intracellular ROS generation intensity, (**B**) Intracellular ROS generation fold changes, (**C**) nuclear condensation intensity, (**D**) nuclear condensation fold changes, (**E**) mitochondrial content intensity, and (**F**) mitochondrial content fold changes, graphs. Every datum shown is the mean ± SD of three experiments.

In an aerobic organism, ROS generation is a normal event in healthy cells. The ROS generation ability and oxidant damage were the parameters used to study F-AgNPs’ potential [56]. ROS generation creates oxidative stress which is induced by metallic nanoparticles during redox cycling; this oxidative stress the disturbs redox equilibrium and affects the defense mechanism of the cell [57]. F-AgNPs damaged cell DNA and altered proteins which are responsible for excess growth, mutagenesis and apoptosis in A549 cells [58]. DCFHDA fluorogenic staining was used to measure the intracellular ROS generation in F-AgNPs-treated A549 viable cells at IC_50_ (Figure 6A,B). The level of ROS generated in F-AgNPs-treated A549 cells was proportional to the fluorescence intensity, and it was extremely high in treated cells compared to normal ones. The quantitative estimation of the intracellular ROS level in F-AgNPs-treated A549 cells demonstrated by the fluorescence intensity was found to be 61.5 a.u. (Figure 7A), and the fold change was 2.57 times (Figure 7B) as compared to the control. The overproduction of ROS and oxidative stress in F-AgNPs-treated A549 cells were possibly involved in cytotoxicity.

### 4.6. F-AgNPs Induced Nuclear Morphological Changes in A549 Cells

The cellular uptake and internalization, along with the apoptotic properties of F-AgNPs, were revealed by using DAPI staining. The F-AgNPs (at IC50 concentration)-treated cells were found to have changes in cellular morphology, reduced chromatin, and expanded cell membrane penetrability which created dark blue nuclei [59]. However, untreated control cells did not disclose any significant fluorescence (Figure 6C,D). The quantitative estimation of the nuclear degradation level in F-AgNPs-treated A549 cells demonstrated by the fluorescence intensity was found to be 79.4a.u. (Figure 7C), and the fold change was calculated to be 5.05 (Figure 7D) as compared to the control. Additionally, some other characteristics of apoptosis—a change in nuclear morphology, condensed nuclei, and membrane disruption—were also observed in F-AgNPs-treated A549 cells. These changes in nuclear morphology were due to the caspase cascade activation, which sliced the substrate at a specific site that is responsible for DNA activation.

### 4.7. F-AgNPs Interrupts Mitochondrial Membrane Potential (ΔΨm) in A549 Cells

**Figure 8:**
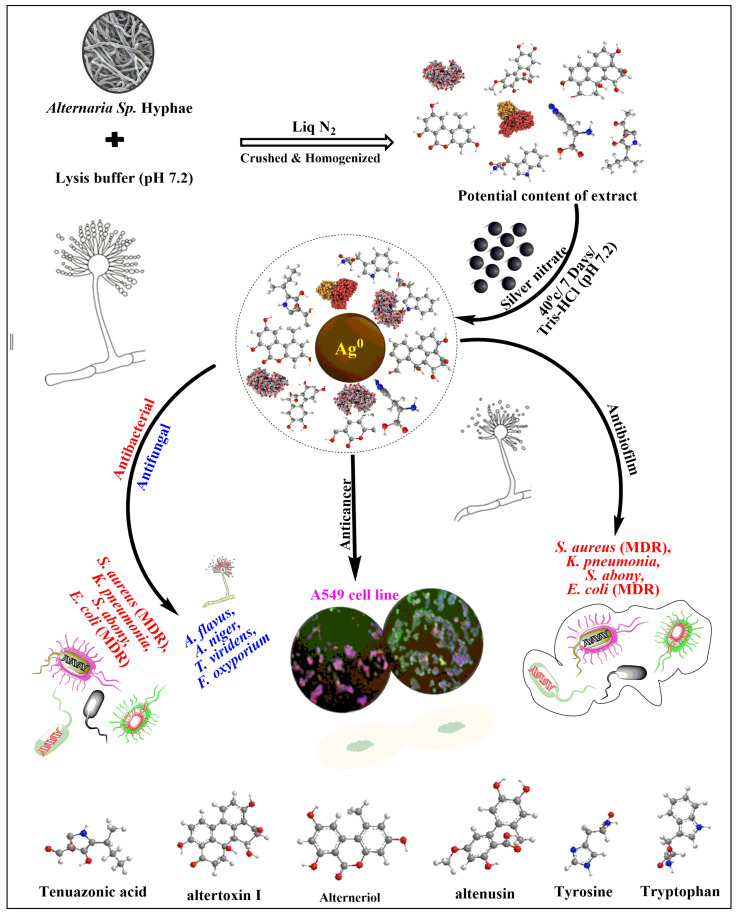
Schematic representation of F-AgNPs’ synthesis and their antibacterial, antifungal, anticancer, and antibiofilm properties.

A reduction in mitochondrial membrane potential is the key to mitochondrial apoptosis [60]. Mito Tracker Red CMX Ros dye was used to stain A549 cells treated with F-AgNPs at IC_50_ to detect the change in ΔΨm disruption. The fluorescence intensity of F-AgNPs-treated A549 cells was found to be decreased as compared to untreated control cells (Figure 6E,F). The quantitative estimation disruption of ΔΨml level in F-AgNPs-treated A549 cells was demonstrated by the fluorescence intensity, which was found to be 15.19 a.u. (Figure 7E), and the fold change was estimated to be 0.19 (Figure 7F) compared to the control. The given silver nanoparticles (F-AgNPs) offer great potential for medical research, including microbiology and cancer biology. The medicinal role of silver has been exploited since ancient times, and its uses have been increased exponentially (Figure 8). Naturally derived silver nanoparticles, including F-AgNPs, could be tested in ointments and creams to annihilate the bacterial infections in open wounds and burns. F-AgNPs-coated medical devices and implants could be manufactured to provide complete protection against any secondary infection. Moreover, silver has been utilized in many consumer products, including colloidal silver gel and silver-embedded fabrics in sporting equipment. F-AgNPs can now be proposed as a very strong candidate against bacterial growth, bacterial biofilms, and cancer cell propagation. The toxicity assessment of nanomaterials is a must before designing the applications of the same. For that, quantitative structure–activity relationship/quantitative structure–property relationship (QSAR/QSPR) has shown its accuracy and offers viable solutions to dictate the associated risk assessments in plants and animals [61]. Moreover, the concept of nano-QSPR and nano-QSAR are comparatively innovative approaches that are beginning to be considered to overcome the current technical limitations in the knowledge of whole nanomaterial behavior. 

## 5. Conclusions

Now, it can be concluded that the biogenic nanoparticles we used have several medicinal properties from the source(s) used in their biosynthesis that are distinct from the intrinsic properties of metal nanoparticles. The F-AgNPs clearly showed antibacterial, antibiofilm, and anticancer properties which were also shown by *Alternaria sp*. Therefore, the metal nanoparticles received several metabolites, enzymes, and proteins from their redox source and mimic their properties, though in fact outdoing the parent source due to the accumulation of the compounds at the nanoscale, which allows them to act synergistically. This study could be extended by finding the mechanisms of action for these F-AgNPs.

## Data Availability

Not applicable.
